# The Influence Mechanism of Learning Orientation on New Venture Performance: The Chain-Mediating Effect of Absorptive Capacity and Innovation Capacity

**DOI:** 10.3389/fpsyg.2022.818844

**Published:** 2022-05-30

**Authors:** Yanling Yang, Yanling Zheng, Guojie Xie, Yu Tian

**Affiliations:** ^1^Business School, Shandong Normal University, Jinan, China; ^2^School of Management, Guilin University of Aerospace Technology, Guilin, China; ^3^School of Management, Guangzhou University, Guangzhou, China; ^4^School of Business, Sun Yat-sen University, Guangzhou, China

**Keywords:** learning orientation, absorptive capacity, innovative capability, new venture performance, chain-mediating effect

## Abstract

New ventures have stronger learning motivation but higher failure rates. In the era of the digital economy, it is necessary to clarify whether and how learning orientation gives scientific guidance for new ventures. We developed a chain multiple intermediary model following the paradigm of “orientation → capability → performance,” which was empirically analyzed using data from 214 Chinese new ventures. The results show that learning orientation not only has a direct positive impact on new venture performance (NVP) but also has an indirect positive effect through the chain-mediating effect of absorptive capacity and innovation capacity. The study advances theoretical understanding of the effect and path of learning orientation on NVP, fosters in-depth research on organizational learning and dynamic capability, and provides targeted organizational learning solutions for new ventures in emerging economies.

## Introduction

With developments in the digital economy and gradual formation of the big data environment, the increasing time-sensitivity of knowledge, shortened innovation cycle of products, and scarcity of customer attention lead to fierce market competition that constantly eroded the existing advantages of enterprises. In fact, the significance of learning for organizational development has been highly recognized by researchers, but we still need to examine the subject of how organizations conduct effective learning and how the impacts of learning convert into business performance (Bašić, 2021). Especially for new ventures lacking early resource accumulation and past transaction records, they often face severe challenge of “new weakness,” resulting in stronger motivation to learn, eager to embed themselves in external markets through organizational learning, so as to obtain resource support, enhance knowledge spillover, and overcome resource constraints (Chung et al., 2019; Bašić, 2021). However, compared with mature firms, new ventures lack both original knowledge accumulation and established experience to learn from. There are many cases of organization learning failure, and new ventures demand scientific advice on advanced learning approaches.

Learning orientation has received considerable attention in recent years as a kind of critical learning concept. Scholars have explored the relationship between learning orientation and enterprise performance (Calantone et al., 2002; Sheng and Chien, 2016; Honig and Hopp, 2019). Existing studies that focuses primarily on mature enterprises in developed countries and ignores new ventures in emerging economies (Tuomisalo and Leppäaho, 2019), is unable to provide effective solutions for new ventures in emerging economies due to a lack of understanding about their learning characteristics. Furthermore, existing research is limited to the effect of learning orientation on firm performance, and findings vary significantly due to a lack of in-depth examination of the specific pathways linking learning orientation and firm performance (Honig and Hopp, 2019), further undermining the theoretical guidance for practice. As a result, the effect and mechanism of learning orientation on new venture performance (NVP) largely unresolved.

According to organizational learning and dynamic capabilities theories, possessing resources is a necessary but not sufficient condition for a firm’s success; mastering the dynamic capabilities of resource transformation is where a firm’s core competencies lie. Absorptive capability and innovative capability have gained much attention as critical dynamic capabilities (Ince et al., 2016). Surprisingly, the logical connection between absorptive and innovative capabilities has rarely been discussed (Limaj and Bernroider, 2019). It is still unknown what function each plays in the complicated process of changing a firm’s strategic orientation into performance, and what link exists between them. Our research indicates that learning orientation assists new ventures in appreciating knowledge, absorption capability assists in accelerating knowledge transformation, and innovation capability assists in applying new knowledge, all of which contribute to the qualitative transformation of “orientation” into “capability” and finally into “performance.” Therefore, it is quite likely that absorptive capability and innovative capability play critical mediating roles in the relationship between learning orientation and NVP. Research is necessary to demonstrate the relationship.

To summarize, the central concerns to be addressed in this study are whether and how learning orientation impacts the performance of new ventures. We constructed a chain multiple mediation model following the research paradigm of “oriented → capability → performance.” Learning orientation was used as the independent variable, absorptive capacity and innovation capacity as double mediating variables, and NVP as the outcome variable. Then, to answer the following two questions, an empirical analysis was conducted utilizing data from 214 Chinese new ventures: First, what effect does learning orientation have on NVP? to find out how learning orientation affects NVP; second, how does learning orientation affect NVP? To uncover the mechanisms through which learning orientation influences NVP, with a focus on the chain-mediating role of absorptive capacity and innovative ability.

## Literature Review and Hypothesis

### Learning Orientation and New Venture Performance

Learning orientation refers to the attitude and tendency of an organization to attach importance to learning and regard it as a valuable activity. By integrating learning into organizational culture, learning orientation can influence employees’ behaviors and promote continuous learning to improve organizational competitiveness. Learning orientation usually comprises three dimensions: learning commitment, shared vision, and open mind (Sinkula et al., 1997). Learning commitment refers to how an enterprise regards learning as an essential aspect of improving the organization and is considered one of the most important values of an enterprise. Shared vision refers to the construction of a common vision within the organization so that the employees can realize their responsibility for the future development of the enterprise. Open mind means that the enterprise can question the value of knowledge and dare to break the mold of creative learning, demonstrating the critical learning method of the enterprise.

At present, numerous studies have explored the influence of learning orientation on enterprise performance. Some studies have found that learning orientation positively influenced enterprise performance through the entire mediating effect of organizational learning (Real et al., 2014), or directly and positively impacted company innovation, but is positively moderated by the firm’s business model (Rahab, 2012). Some have argued that learning orientation did not directly affect enterprise performance but instead played a positive moderating role between innovation capability and service innovation performance (Pesämaa et al., 2013). The different conclusions suggest a complex mechanism between learning orientation and enterprise performance that requires further exploration.

With developments of the digital economy, the characteristics of organizational structure platform, relationship network, and context ecology have become more apparent, causing profound effects on organizational learning environment, learning mode, and learning effect. As a result, new ventures in the new era urgently need scientific guidance on learning orientation. Learning orientation directs new ventures toward meeting their learning commitments through active use of convenient social media tools to interact with other stakeholders closely, absorb external information extensively and build a good social relationship network to help achieve accessibility to vital resources and resolve resource constraint problems (Iyer et al., 2019). Utilizing the advantages of a small organizational structure and the impact of charismatic leaders effectively would assist new ventures in developing a clearer picture of the organization’s shared vision, stimulating employees’ sense of ownership, enhancing organizational cohesion, and improving corporate reputation and appeal (Johansson et al., 2019). New ventures are encouraged to adopt an open mind, carefully listen to customer opinions and suggestions, understand better consumer needs and market trends, and involve customers in product innovation for value co-creation to improve enterprise innovation and market acceptance for new products (Xie et al., 2021). Based on the discussion, the hypothesis is proposed as follows:

*H1*: Learning orientation has a direct positive effect on new venture performance.

### Absorptive Capacity in the Relationship Between Learning Orientation and New Venture Performance

Absorptive capacity refers to the norms and procedures by which a business obtains, digests, transforms, and applies knowledge in order to enhance the organization’s dynamic capacities (Zahra and George, 2002). Its four main dimensions are acquisition capability, absorption capability, transformation capability, and utilization capability (Flatten et al., 2011). Acquisition capability refers to the enterprise’s ability to identify and acquire knowledge inside and outside the industry, while absorption capability refers to the circulation of acquired knowledge inside and outside the enterprise. Transformation capability refers to the processing ability of knowledge, and utilization capability refers to the enterprise’s ability to apply new knowledge after transformation.

Absorptive capacity theory has received considerable attention as a viable perspective and way of thinking about enterprise performance, for integrating resource-based theory and dynamic capability theory, and for complementing and contributing to hot topics, such as organizational learning, strategic management, and knowledge management (Lewin et al., 2011). Studies have been conducted to analyze the antecedent and consequence variables of absorptive capacity. For example, a number of studies have explored the influence of characteristics of knowledge (Flor et al., 2018), enterprise network (Dos Santos et al., 2021), and internal mechanism of enterprises (Apriliyanti and Alon, 2017) on absorption capacity and the influence of absorptive capacity on knowledge transfer (Schweisfurth and Raasch, 2018), competitive advantage (Solís-Molina et al., 2018), and enterprise performance (Kale et al., 2019).

The digital economy has resulted in big data with large numbers, wide varieties, rapid updates, and increased complexities. The capability to process, analyze, and synthesize scattered and disorganized large amounts of data based on the needs of enterprise development to form orderly, high-quality referable information resources is the core value of big data (Iosifidis et al., 2017). In comparison to mature businesses, new ventures lack sophisticated information processing systems and standardized procedures, making it difficult to share the business value created by big data equally. However, new ventures have the advantage of being small and adaptable, unconstrained by prior experience, and more responsive to new ideas, which fits well with the inherent requirement of learning-oriented open-mindedness. Learning orientation encourages new ventures to put their commitment to learning into practice and to acquire new external information on a regular basis through continuous learning, which can assist them in effectively resolving the problem of knowledge time-sensitivity and enhancing their knowledge acquisition capability (Riikkinen et al., 2017). Learning orientation encourages new ventures to be open-minded, to challenge the worth of knowledge, and to respond appropriately to external criticism, all of which can assist them in efficiently resolving the problem of one-sided knowledge absorption and enhancing their knowledge system (Kale et al., 2019). Additionally, learning orientation encourages new ventures to develop a shared vision and supports them in processing information holistically, avoiding problems, such as disordered knowledge absorption, isolation, duplication, and redundancy. Therefore, the following hypothesis is proposed:

*H2*: Learning orientation has a direct positive effect on absorptive ability.

The advancement of Internet technology and the widespread use of mobile terminal devices, such as smartphones, have resulted in the emergence of a variety of transactions, such as virtual communities, Internet banking, self-service terminals, and online supermarkets, all of which have generated a large amount of unstructured and semi-structured data and placed increased demands on enterprises’ data absorption capacity. In today’s severe market rivalry, only by consistently developing their ability to acquire knowledge and collecting comprehensive external information can start-up firms avoid missing business chances.

The development of Internet technology and the popularity of mobile terminal devices, such as smartphones, have given rise to a variety of transactions, such as virtual communities, Internet banking, self-service terminals, and online supermarkets, which have generated a large amount of unstructured and semi-structured data and put forward higher requirements on the absorption capacity of enterprises. In the fierce market competition, only by continuously improving knowledge acquisition ability and extensively collecting external information can start-up enterprises avoid missing business opportunities. They can obtain useful information only by effectively cleaning massive data, such as correlation analysis of products and services, social network analysis, and user behavior analysis; extraction of correct information about consumers’ fundamental motivations, consumer preferences, and potential demands, as well as internalization of external tacit knowledge into new company knowledge, can offer valuable references for enterprise decision-making (Pesämaa et al., 2013). Finally, new ventures should also combine the transformed new knowledge with their existing technologies and apply them to their production practices in a timely manner so that they can really bring good benefits to the enterprises (Siachou et al., 2021). Thus, it is evident that only strong absorptive capacity enables new enterprises to translate abundant external data into unique internal intellectual support, which is then translated into corporate performance. Therefore, the hypothesis is proposed as follows,

*H3*: Absorptive capacity has a direct positive impact on new venture performance.

To summarize, learning orientation encourages start-up businesses to prioritize organizational learning, advocates for businesses to abandon passive learning postures and adopt active and conscious behaviors, widely absorb diverse external knowledge to avoid thinking anchored, and actively engage in open thinking to think outside the box (Riikkinen et al., 2017), thereby assisting enterprises in developing an open, multi-channel, interactive knowledge exchange mechanism, and accelerate enterprises’ acquisition of knowledge. Furthermore, good absorptive capacity can assist start-up businesses in acquiring rich external information in a timely manner, refining it precisely, efficiently transforming knowledge, and applying it to production practices in a timely manner, thus providing continuous intellectual support for the development of new products and services and assisting businesses in achieving good performance (Schweisfurth and Raasch, 2018). Therefore, the hypothesis is proposed as follows:

*H4*: Absorptive capacity plays a mediating role between learning orientation and new venture performance.

### The Role of Innovation Capability Between Learning Orientation and New Venture Performance

Innovation capability refers to the efficiency of a particular output brought by an enterprise’s input of specific innovation resources, including incremental innovation and breakthrough innovation (Saunila, 2020). Incremental innovation refers to the organization using existing technology to make minor improvements to existing products or services, which is a “perfect” steady innovation. Breakthrough innovation refers to the use of transformative technologies by organizations to develop new products or services, which is a “subversive” thorough innovation (Beatriz Forés, 2016). Organizational learning (Sheng and Chien, 2016), technical research and development (Erdal and Göçer, 2015), absorptive capacity (Khan et al., 2019), and relational capital (Sulistyo and Siyamtinah, 2016) have attracted more attention in studies on the antecedent variables of innovation capability. Numerous studies analyzing the consequences of innovation capability have focused on its effect on the enterprise’s performance (Lennerts et al., 2020). However, analyzing innovation capability based on the learning orientation perspective has largely been overlooked. As one of the important bridges between knowledge and performance, the specific mechanism of innovation capability needs to be further clarified.

The broadening of the “Internet +” concept has created a slew of new business models, providing consumers more options and making them more selective. As a result, customer needs have increasingly diversified and personalized. Therefore, identifying and addressing customers’ potential needs present great challenges to the innovation capability of new ventures. In comparison to mature firms, new ventures are more circumspect in selecting their innovation strategies, as competitive strength affects market power, and new ventures must tread carefully under the simultaneous pressures of “new weakness” and “organizational legitimacy.” Under the duress of “innovation” and “legitimacy,” new companies must delicately balance “innovation” and “legitimacy” in order to avoid a survival dilemma (Saunila, 2020). New ventures are guided by learning orientation to engage in critical learning and open thinking. On the one hand, actively learning from competitors enables them to more objectively assess their own capabilities and forecast market trends, offering a more scientific basis for their innovation decisions (Pesämaa et al., 2013). On the other hand, learning from customers with an open mind helps organizations to obtain valuable tacit knowledge of customers and better understand their consumption preferences and potential demands, which helps to improve the goal-setting and success rate of their innovation (Sheng and Chien, 2016; Johansson et al., 2019; Xie et al., 2021). Based on these arguments, the following hypothesis is proposed:

*H5*: Learning orientation has a significant positive influence on innovation capability.

The positive influence of innovation on corporate performance is highly recognized. Incremental innovation by new ventures to develop and refine existing products not only helps firms save R&D expenditure and decrease R&D time, but also lowers the market entry barrier for new products and services, thereby helping firms to swiftly achieve benefits at lower cost and less risk (Saunila, 2020). While the risk and cost of breakthrough innovation are generally high, when successful, it can frequently result in excessive profits or even restructure an industry by upending established market patterns (Sheng and Chien, 2016). Implementing a dual innovation balance strategy can prompt new ventures to comprehensively consider the exclusivity and sharing of resources required for incremental and breakthrough innovation activities, allocate scarce and redundant resources more scientifically, actively coordinate the competitive relationship of dual innovation, and deeply explore the synergistic effects of dual innovation, ultimately improving the performance of new ventures by increasing resource utilization and innovation efficiency. Therefore, the following hypothesis is proposed:

*H6*: Innovation capability has a direct positive impact on new venture performance.

In short, practicing learning orientation can assist new ventures in dialectically accepting external novelties and new ideas, arousing their innovation consciousness, motivating employees’ innovation activity, and therefore enhancing their innovation capabilities. Additionally, a strong innovation capability enables businesses to better understand possible customer needs, properly forecast market trends, and respond quickly to market changes, hence improving product sales and market share. Therefore, the following hypothesis is proposed:

*H7*: Innovation capability plays a mediating role between learning orientation and new venture performance.

### Absorptive Capacity and Innovative Capability

Absorptive capacity and innovation capacity are important dynamic capabilities of enterprises. However, few studies have analyzed their relationship, and there remains a lack of theoretical analysis and empirical research (Yang and Tsai, 2019). This study believes that good absorptive capacity is intended to help new ventures accelerate the accumulation and updating of knowledge to improve the response ability to the external market and emphasize the cultivation of internal competitiveness of new ventures. Excellent innovation capability is the concrete practice of the enterprise’s new knowledge, showing the external expansion of new ventures. The former provides effective information reference and intellectual support for the latter and is the source of the latter’s motivation, while the latter is the application and practice of the former and the practical test of the former. The two work together to improve NVP.

Good absorption capacity can provide timely, comprehensive, and accurate external information for new ventures, accelerate the circulation and sharing of information and knowledge within new ventures, increase knowledge accumulation, improve knowledge structure, promote knowledge spillover, and provide strong intellectual support for new ventures to carry out innovation. Thus, the innovation capability and efficiency of new ventures can be improved (Schweisfurth and Raasch, 2018). Furthermore, excellent innovation ability urges new ventures to constantly develop new technologies and processes, introduce advanced concepts, launch new products and services, consolidate the original market and explore new markets, thus improving sales and corporate reputation and generating tangible benefits (Khan et al., 2019). Based on the discussion, the following hypotheses are proposed:

*H8*: Absorptive capacity has a direct positive impact on innovation capacity.

*H9*: Absorptive capacity and innovative capacity together provide a mediating role between learning orientation and performance of new ventures.

**Figure 1:**
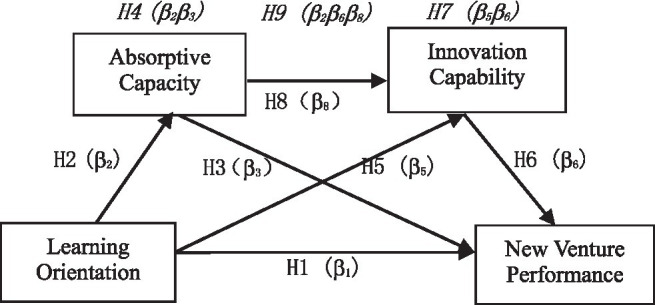
Research model. Note: Italicized terms indicate mediating effects.

The hypothesis model in this study is a chain multiple mediation model using learning orientation as the antecedent variable, absorptive capacity and innovation capacity as the mediators, and new enterprise performance as the outcome variable (see Figure 1). There are three intermediary paths: (1) H_4_: Learning orientation → absorptive capacity → NVP (*β*_2_*β*_3_); (2) H_7_: Learning orientation → innovative capacity → NVP (*β*_5_*β*_6_); and (3) H_9_: Learning orientation → absorptive capacity → innovative capacity → NVP (*β*_2_*β*_6_*β*_8_).

## Research Methodology

### Scale Design

Questionnaire design mainly refers to relevant scales previously developed and tested. To quantify and analyze the responses, a 5-point Likert scale was used. Learning Orientation (LO) scale is based on Sinkula et al. (1997) and includes three dimensions (18 items in total): learning commitment (6 items), shared vision (6 items), and open mind (6 items). Absorptive Capacity (AC) scale is based on Zahra and George (2002) and Flatten et al. (2011) and comprises four dimensions (14 items in total): acquisition ability (3 items), digestion ability (4 items), transformation ability (4 items) and utilization ability (3 items). Innovation Capability (IC) scale is based on Subramaniam and Youndt (2005) and Koberg et al. (2003) and has two dimensions (ten items in total): progressive innovation capability (5 items) and breakthrough innovation capability (5 items). NVP scale is based on Li and Zhang (2007) and is composed of return on sales, return on assets, return on investment, market share growth, sales growth, profit growth, cash flow, overall corporate reputation, and overall operating efficiency. Previous studies have suggested that the scale of the enterprise (with the number of employees as the substitution variable), the nature of the enterprise, and industry type may affect enterprise performance. Since these factors are not the focus of this study, thus were treated as control variables in the analysis.

### Data Collection

Data was primarily collected by checking enterprise yellow pages, contacting firms participating in school–enterprise collaboration, interviewing students from executive training courses organized at universities, and handing out questionnaires on the spot between June 2021 and August 2021. The surveyed companies were new ventures in China’s Pearl River Delta region (in operation for at most 8 years). Out of the 500 questionnaires sent out, 256 were retrieved and 214 were determined to be valid (43 incompleted ones rejected), with an effective recovery rate of 42.80%. Table 1 summarizes the descriptive statistics of the businesses surveyed in this study.

**Table 1:** Descriptive statistics of the samples.

Enterprise character	Count	Proportion (%)	Enterprise size	Count	Proportion (%)	Industry type	Count	Proportion (%)
State-owned	31	14.49	<100	105	49.07	Manufacturing	86	40.19
Private	104	48.60	100–499	89	41.59	Services	84	39.25
Joint-stock	35	16.36	500–999	12	5.61	Emerging industries	31	14.49
Sino-foreign joint	38	17.76	1,000–4,999	5	2.34	Social Welfare	5	2.34
Other	6	2.80	>5,000	3	1.4	Other	8	3.74
Total	214	100	Total	214	100	Total	214	100

## Data Analysis and Hypothesis Testing

### Common Method Bias Analysis

We used process control and statistical testing methods to minimize common method bias (CMB). Three questionnaire formats were employed: online version, paper version, and electronic version. To enhance the validity of the responses, the questionnaires were kept anonymous, with some negatively worded items, and respondents were encouraged to answer the questions collectively. To assess the degree of common method bias, the Harman single-factor method was deployed. It revealed nine factors with eigenvalues larger than 1, accounting for 68.69% of the variance explained, with the first principal component accounting for 33.57% of the variance. This suggests that the common method bias in this study is minor and would not significantly affect the relationships between variables.

### Reliability and Validity Analysis

The confirmatory factor analysis was performed using AMOS23.0 software, and the summary of results is presented in Table 2. The item factor load values had a minimum of 0.530 and a maximum of 0.885, which are within the tolerable range of 0.500–0.950.

**Table 2:** Results of the confirmatory factor analysis.

Items	Factor loading	Items	Factor loading	Items	Factor loading	Items	Factor loading
Learning orientation		LO32	0.785	AC32	0.770	IC23	0.844
LO11	0.736	LO33	0.810	AC33	0.789	IC24	0.849
LO12	0.702	LO34	0.818	AC34	0.818	IC25	0.798
LO13	0.733	LO35	0.833	AC41	0.729	New venture performance	
LO14	0.750	LO36	0.779	AC42	0.845	NVP1	0.774
LO15	0.789	Absorptive capacity		AC43	0.789	NVP2	0.793
LO16	0.747	AC11	0.754	Innovation capability		NVP3	0.725
LO21	0.741	AC12	0.885	IC11	0.843	NVP4	0.683
LO22	0.820	AC13	0.684	IC12	0.880	NVP5	0.776
LO23	0.799	AC21	0.825	IC13	0.826	NVP6	0.771
LO24	0.795	AC22	0.780	IC14	0.876	NVP7	0.749
LO25	0.762	AC23	0.803	IC15	0.767	NVP8	0.779
LO26	0.716	AC24	0.702	IC21	0.784	NVP9	0.766
LO31	0.530	AC31	0.752	IC22	0.818		

For the reliability test, the Cronbach coefficient was used. The Cronbach coefficients for all variables were greater than 0.8, as shown in Table 3, indicating good reliability. The AVE values for all the variables were greater than 0.5, and the CR values were greater than 0.6, suggesting strong convergence validity. Furthermore, all the square root AVE values were larger than the correlation coefficient across factors, indicating excellent discriminant validity.

**Table 3:** Results of the reliability and validity analysis.

Variable	*α*	Mean	SD	CR	AVE	LO	AC	IC	NVP
LO	0.945	3.708	0.621	0.951	0.564	**0.751**			
AC	0.920	3.621	0.644	0.955	0.612	0.356^**^	**0.782**		
IC	0.945	3.529	0.711	0.957	0.688	0.577^**^	0.386^**^	**0.829**	
NVP	0.826	3.747	0.633	0.924	0.575	0.548^**^	0.484^**^	0.646^**^	**0.798**

** *p* < 0.01.

Square root of AVEs in boldface on the diagonal of the matrix.

### Hypothesis Testing

**Figure 2:**
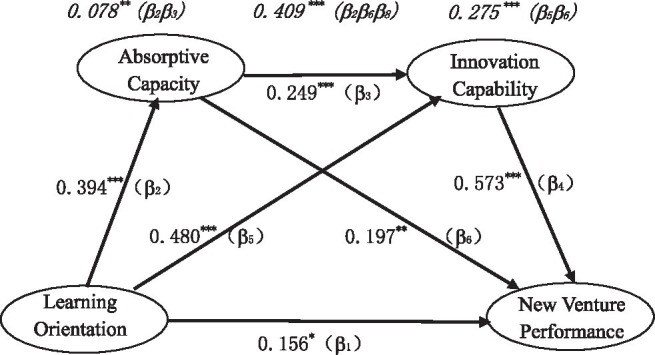
The Output of the hypothesis model. Note: Italics indicate mediating effects. *p < 0.05, **p < 0.01, and ***p < 0.001.

The hypotheses were tested using the non-parametric percentile Bootstrap method with bias correction. The following steps were employed: (1) In the Bootstrap program, the number of repeated samples was set to 5,000, and the confidence interval was set to 95%. (2) The Bootstrap program generated 5,000 effect estimates, which were then automatically sorted. (3) The value for the 95% confidence interval was then evaluated. If 0 is included, the effect is not significant; if 0 is not included, the effect is significant. The output of the hypothesis model is shown in Figure 2, and the hypothesis test results are shown in Table 4.

**Table 4:** Results of the hypothesis testing.

Influence path	Direct effect	95% confidence interval	Indirect effect	95% confidence interval
LO → NVP	0.156^*^	[0.003,0.305]	0.409^***^	[0.288,0.557]
LO → AC	0.394^***^	[0.244,0.526]	–	–
AC → NVP	0.197^**^	[0.062,0.332]	0.143^**^	[0.062,0.240]
LO → IC	0.480^***^	[0.296,0.638]	0.098^***^	[0.038,0.186]
IC → NVP	0.573^***^	[0.428,0.715]	–	–
AC → IC	0.249^***^	[0.103,0.395]	–	–

* *p* < 0.05,

** *p* < 0.01, and

*** *p* < 0.001.

#### Direct Effect Test

(1) In the influence path from learning orientation to NVP, the 95% confidence interval for direct effect is [0.003,0.305], excluding 0, and the standardized path coefficient *β*_1_ is 0.156 (*p* = 0.031). The results suggest that learning orientation has a significant direct positive effect on NVP. Hypothesis H1 has been verified. (2) In the influence path from learning orientation to absorptive ability, the 95% confidence interval for the direct effect is [0.244,0.526], excluding 0, and the standardized path coefficient β_2_ is 0.394 (*p* < 0.001). This indicates that learning orientation has a significant direct positive effect on absorptive ability. Hypothesis H2 has been verified. (3) In the influence path from absorptive capacity to NVP, the 95% confidence interval for the direct effect is [0.062,0.332], excluding 0, and the standardized path coefficient *β*_3_ is 0.197 (*p* = 0.003). The results suggest that absorptive capacity has a significant direct positive effect on NVP. Hypothesis H3 has been verified. (4) In the influence path from learning orientation to innovation capability, the 95% confidence interval for the direct effect is [0.296,0.638], excluding 0, and the standardized path coefficient *β*_5_ is 0.480 (*p* < 0.001). The results indicate that learning orientation has a significant direct positive effect on innovation capability. Hypothesis H5 has been verified. (5) In the influence path from innovation capability to NVP, the 95% confidence interval for the direct effect is [0.428,0.715], without 0, and the standardized path coefficient *β*_6_ is 0.573 (*p* < 0.001). This means that innovation capability has a significant direct positive effect on NVP. Hypothesis H6 has been verified. (6) In the influence path from absorption capacity to innovation capacity, the confidence interval for the direct effect is [0.103,0.395], without 0, and the standardized path coefficient *β*_8_ is 0.249 (*p* < 0.001). The findings suggest that absorptive capacity has a significant direct positive effect on innovation capacity. Hypothesis H8 has been verified.

#### Indirect Effect Test

The results suggest that the standardized path coefficients of direct effects *β*_1_, *β*_2_, *β*_3,_ *β*_5_, *β*_6_, and *β*_8_ are all significant. Moreover, in the influence path from learning orientation to NVP, the 95% confidence interval for indirect effect is [0.288,0.557], which does not contain 0. This means that the indirect effect between learning orientation and NVP is significant. In addition, in the influence path from learning orientation to innovation capacity, the 95% confidence interval for indirect effect is [0.038, 0.186], excluding 0, indicating that the indirect effect between learning orientation and innovation capacity is significant. In the influence path from absorptive capacity to NVP, the 95% confidence interval for indirect effect is [0.062, 0.240], excluding 0. This suggests that the indirect effect between absorptive capacity and NVP is significant.

In conclusion, absorptive capacity and innovation capacity jointly play a chain-mediating role between learning orientation and NVP. The total mediating effect of the two is 0.409 (*β*_2_*β*_3_ + *β*_5_*β*_6_ + *β*_2_*β*_6_*β*_8_), accounting for 72.39% of the total effect. The mediating effect of absorption capacity alone was 0.078 (*β*_2_*β*_3_), accounting for 13.74% of the total effect. Hypothesis H4 has been verified. The mediating effect of innovation capacity alone was 0.275 (*β*_5_*β*_6_), accounting for 48.68% of the total effect. Hypothesis H7 has been verified. The chain-mediating effect of absorptive capacity and innovation capacity was 0.056 (*β*_2_*β*_6_*β*_8_), accounting for 9.95% of the total effect. Hypothesis H9 has been verified.

## Conclusion and Discussion

### Conclusion

We created a hypothesis model and conducted empirical analysis on 214 Chinese new ventures using the “orientation-capability-performance” paradigm. The findings indicate that learning orientation has a positive effect on NVP *via* a variety of mechanisms, including (1) learning orientation → NVP, (2) learning orientation → absorptive capacity → NVP, (3) learning orientation → innovation capacity → NVP, (4) learning orientation → absorptive capacity → innovative capacity → NVP. The findings indicate that learning orientation has a direct and positive impact on NVP, as well as an indirect positive effect *via* the partly mediating effect of absorptive and inventive capacity. Absorptive capacity and innovation capacity work in tandem to mediate the relationship between learning orientation and NVP, with a chain-mediating effect of 0.409, which accounts for 72.39 percent of the total effect and explains the majority of the variance between learning orientation and NVP.

### Discussion

To begin, learning orientation has a significant positive impact on NVP, and exercising learning orientation in the digital economy enables new ventures to overcome their “new weakness.” Existing organizational learning research has concentrated on mature firms in developed nations in the hope of eliciting organizational learning experiences and strategies through successful case studies. However, because the start-up environment and resource endowments of new ventures in emerging economies are significantly different from those of mature firms, it is difficult to copy or replicate mature firms’ successful experiences, and organizational learning must be emphasized to achieve sustainable development (Limaj and Bernroider, 2019). This study empirically examines the significant positive effect of learning orientation on NVP, implying that start-up enterprises elevate organizational learning to a strategic level, integrate continuous learning into corporate culture, quickly overcome the disadvantage of insufficient internal knowledge accumulation through external knowledge acquisition, and exercise diligence in thinking and questioning. All of these activities contribute to the development of sophisticated knowledge systems and an enabling environment for innovation, to the ongoing improvement of the ability to integrate resources, and to the adaptation of the market and organizational environment.

Second, absorption significantly mediates the relationship between learning orientation and new enterprise performance, and having good absorption capacity is an important strategy for new companies to establish core competitiveness. While the critical function of absorptive capacity has been examined in detail in previous research, there is still considerable room for investigating how to improve organizations’ absorptive capacity (Haryanti and Subriadi, 2022). To ensure equal access to the business value of big data in the digital economy, it is necessary for new ventures with limited knowledge accumulation and learning capabilities to practice learning orientation. This includes encouraging employees to think critically in order to continuously expand the scope of knowledge acquisition, guiding employees toward dialectical absorption in order to continuously improve the efficiency of knowledge transformation, and actively supporting employees in order to establish a common vision. Thus, the strategic deployment (learning orientation) of the organization can be translated into tactical capabilities (absorption capacity), which will ultimately contribute to the new enterprise’s performance enhancement.

Third, Innovation capability plays a significant role in mediating the relationship between learning orientation and NVP, meaning that a high innovation capability is necessary to transform knowledge into performance. The emphasis of innovation capability research is gradually changing away from “impact utility” and toward “strategy choice.” With the development of the digital economy, the emergence of new business models, and the acceleration of product and service iterations, the only way for new ventures to qualify for market entry is through innovation. The finding suggests that new ventures should adhere to the spirit of learning, pursue a learning attitude, and adopt a bold, pioneering spirit of enterprise culture. New ventures should improve their employees’ sense of responsibility, motivating them to take initiatives and actively contribute their ingenuity to optimize enterprise innovation. These measures would significantly reduce the costs of enterprise innovation and improve the efficiency of enterprise innovation.

Fourth, absorptive capacity and innovation capability play a complex mediating role between learning orientation and NVP. While scholars have paid considerable attention to absorptive and inventive capabilities as critical dynamic capabilities of businesses, few studies have examined their internal logic in detail (Ince et al., 2016). Based on organizational learning theory and dynamic capability theory, we discovered in this study that absorptive capacity is the driving force behind innovation capability, and innovation capability is the practical test of absorptive capacity, and that they function in concert to improve company performance. The results suggest that new ventures should focus on both absorptive and innovative capacity simultaneously, focusing not only on the acquisition, absorption, and transformation of information but also on using the new knowledge after transformation. Through continuous innovation and improvements, firms would be able to significantly increase performance and provide high-quality customer experience.

### Theoretical Contribution and Practical Implications

Based on organizational learning theory and dynamic capability theory in the context of the digital economy, it is vitally important to examine the impacts and trajectories of learning orientation on the performance of new ventures based on their organizational learning characteristics.

The main contributions and innovation introduced in this study are as follows:

First, the use of Chinese new companies as a sample for empirical study is a valuable addition to the sample of organizational learning studies. As an emerging economy, one of the secrets to China’s remarkable economic development is the upsurge in domestic entrepreneurship and innovation. This study explores the learning features of new ventures in emerging economies and conducts empirical analysis on a sample of 214 start-ups in China’s Pearl River Delta to explore the learning orientation and path of new ventures in an emerging economy in the context of the digital economy era. This study overcomes the limitations of prior organizational learning research that focused on mature companies in developed countries and provides more targeted organizational learning solutions for new ventures in other emerging economies, thereby promoting entrepreneurial activity and contributing to the economy’s balanced development.

Second, defining the effect of learning orientation on NVP provides scientific guidance for startup learning in the new era. The digital economy’s rapid growth and the gradual rise of a big data environment present multiple challenges for organizational learning. Compared with mature, established ventures, new ventures have stronger learning motivation but higher failure rates, suggesting that their organizational learning has distinct characteristics and emphasizing the importance of determining whether learning orientation can give scientific direction for them. Taking into account the strengths and weaknesses of new ventures in the context of the digital economy, the study examines how learning orientation might help overcome “new weaknesses” and “organizational legitimacy” through learning commitment, shared vision, and open-mindedness. It offers a scientific framework for effective learning in the digital economy.

Third, investigating the inherent logic of absorptive and innovative capacity aids in deciphering the “black box” relationship between learning orientation and NVP. Despite the fact that both capabilities are important dynamic qualities of businesses, few studies have thoroughly studied their roles in company growth, their association, and differentiations. Based on the “orientation-competence-performance” paradigm, absorptive capacity has a significant positive impact on innovation capacity, and both capacities play a chain-mediating role in the relationship between learning orientation and NVP. The discovery not only establishes the relationship between absorptive capacity and innovation capability, but also reveals the pathways through which learning orientation influences new ventures, which is significant in dynamic capability research and organizational learning theory.

### Limitations and Future Work

The following limitations in the research should be addressed in the future. First, this study investigates new ventures in China’s Pearl River Delta. The results should be compared with similar studies in other countries or locations. Future research might further validate the findings by focusing on new businesses in other emerging economies, such as India and Brazil, to increase the findings’ external validity. Second, the findings suggest that absorptive capacity and innovation capacity cannot completely explain the impact of learning orientation on NVP. Subsequent studies should explore whether there are still other mediating variables between learning orientation and NVP, such as knowledge integration and resource collocation. Third, this study does not discuss the boundary of learning orientation’s impact on NVP, and lacks situational factor analysis of the relationship between learning orientation and NVP. Future research could examine the effect of environmental dynamics on the link between learning orientation and new business performance in order to better elucidate the learning orientation effect on new business success.

## Data Availability Statement

The raw data supporting the conclusions of this article will be made available by the authors, without undue reservation.

## Ethics Statement

Ethical review and approval were not required for the study on human participants in accordance with the local legislation and institutional requirements. The patients/participants provided their written informed consent to participate in this study.

## Author Contributions

YY and YT: conceptualization. YY: methodology, sofware, writing—original draf preparation, and visualization. YY, YZ, and YT: validation. GX: formal analysis. YY and YZ: investigation. YZ and YT: resources and supervision. YY and YZ: data curation and funding acquisition. YY, YZ, and GX: writing—review and editing. YT: project administration. All authors contributed to the article and approved the submitted version.

## Funding

This work was supported by the Humanities and Social Science Youth Fund project of the Ministry of Education (no. 20YJCZH209) and China Postdoctoral Science Foundation (no. 2019M662435).

## Conflict of Interest

The authors declare that the research was conducted in the absence of any commercial or financial relationships that could be construed as a potential conflict of interest.

## Publisher’s Note

All claims expressed in this article are solely those of the authors and do not necessarily represent those of their affiliated organizations, or those of the publisher, the editors and the reviewers. Any product that may be evaluated in this article, or claim that may be made by its manufacturer, is not guaranteed or endorsed by the publisher.
